# The strongest predictors of compliance with health protocols among marketers and guilds based on the transtheoretical model

**DOI:** 10.1186/s12889-024-19386-w

**Published:** 2024-07-15

**Authors:** Shandiz Moslehi, Asghar Tavan, Sajjad Narimani, Fardin shahbazzadeh, Nadia Sedri, Sama Sabahi

**Affiliations:** 1 Health Management and Economics Research Center, Health Management Research Institute, Iran University of Medical Sciences, Tehran, Iran; 2 Department of Health in Disasters and Emergencies, School of Health Management and Information Sciences, Iran University of Medical Sciences, Tehran, Iran; 3 Health in Disasters and Emergencies Research Center, Institute for Futures Studies in Health, Kerman University of Medical Sciences, Kerman, Iran; 4 Faculty of Management and Medical Information Sciences, Kerman University of Medical Sciences, Kerman, Iran; 5 Department of Nursing and midwifery, School of Nursing, Social Determinant of Health Research Center, Ardabil University of Medical Sciences, Ardabil, Iran; 6 Students Research Committee, Ardabil University of Medical Sciences, Ardabil, Iran; 7 Nursing Research Center, Kerman University of Medical Sciences, Kerman, Iran

**Keywords:** COVID-19 pandemic, Health protocols compliance, Transtheoretical model

## Abstract

**Background:**

Global communication, an integral part of modern life, increases the risk of transmitting infectious diseases to individuals. Based on the transtheoretical model (TTM), this study aimed to identify the most effective factors in adherence to health protocols among marketers and guilds.

**Methods:**

This cross-sectional study was conducted among 400 market sellers and guilds of Ardabil City, Iran, in 2023. The TTM questionnaire was distributed among the participants which included four sub-constructs: (1) stages of change, (2) process of change, (3) self-efficacy, and (4) decisional balance. The data were analyzed using SPSS version 20. One-way ANOVA and linear regression tests were employed to evaluate the prediction of effective factors of the stage transition.

**Results:**

Most participants (63.5%) were between 21 and 40. Most participants (65.5%) were in the passive stages of change (precontemplation, contemplation, and preparation). Pros (β = 0.133, *P* < 0.001) and behavioral processes of change (β = 0.058, *P* < 0.001) were the strongest predictors of the stage of change or improvement of stages of participants’ willingness to follow health protocols.

**Conclusion:**

A correct understanding of the stages of behavior change can strengthen strategies for promoting healthy behaviors. Also, understanding the benefits of healthy behavior means compliance with health protocols and behavioral processes such as stimulus control, reinforcement management, counterconditioning, and self-liberation, along with high self-efficacy, have an impact on improving the stages of behavior change.

## Introduction

Although the World Health Organization has declared that COVID-19 is no longer a “global health emergency,” it emphasizes that it is still a global health threat [1]. A pandemic that affected various components of the worldwide community, including public health, economics, education, and mental health, resulted in more than 7 million deaths [2]. In terms of the number of patients and deaths in the Eastern Mediterranean region, Iran was the leader of the countries severely affected by COVID-19 [3, 4].

During the coronavirus pandemic, healthcare systems in all countries were severely affected. Governments prescribed quarantines and communication restrictions at various intervals, either helpful or ineffective, depending on the circumstances [5]. With the out-of-control spread of COVID-19 and numerous problems in the health service system, individual preventive measures have become one of the most effective ways to prevent the spread of the coronavirus in the world [6]. Personal protective measures (such as wearing a face mask, washing hands, and personal hygiene) and social distancing were among the preventive protocols that helped reduce the risk of infection and control the spread of the disease [7].

Identifying the factors affecting compliance with health protocols for the prevention of COVID-19 is essential for any planning and intervention to maintain community health [8]. Studies conducted during the recent epidemic in Iran have examined a wide range of health issues, including risk perception and risk communication related to the preventive behavior of COVID-19 [7, 9–11].

Various studies using models and theories of behavioral sciences have been conducted worldwide during the COVID-19 pandemic, including the health belief model (HBM), the theory of planned behavior (TPB), the theory of reasoned action, social cognition theory, socio-ecological model, extended parallel process model, and transtheoretical model (TTM). Meanwhile, the HBM and the TPB have been the most frequent, and few studies have been conducted with TTM [12].

One of the main issues neglected in the studies is determining the stages of behavior change in compliance with health protocols and the predictors of stage improvement, such as processes of change and decisional balance. In health education, the fundamental principle is determining the stages of change individuals undergo to tailor the training and achieve optimal results. The transtheoretical model (TTM) consists of four sub-constructs: stages of change, processes of change (cognitive-behavioral), decision-making balance, and self-efficacy. It first assesses the stage of change in individuals’ health behavior and then identifies which sub-construct effectively promotes or reduces the stages of change. The stages of change have two subgroups: the passive stages of change (precontemplation, Contemplation, and Preparation), in which changes have not yet occurred, and the active stages of change (Action and Maintenance), in which behavioral changes have occurred [13–16]. Kamran et al. (2023) found that all dimensions of health-promoting behaviors had a significant relationship with the stages of behavior change, decisional balance, and self-efficacy based on TTM in students [17]. In the study of Nishimoto et al. (2023), the results showed that about a quarter of the participants were in the active stages (action and maintenance) of changing healthy behavior and physical activity during COVID-19 [18]. The results of an online survey in the United States also showed that there are reliable and valid measures of TTM constructs to understand the motivation to receive the Covid-19 vaccine [19].

Although there is little evidence of the use of TTM for preventive behaviors against COVID-19, considering the excellent previous records of this model in predicting health behaviors [20–22]. This study aimed to identify the most effective factors in promoting adherence to health protocols among marketers and guilds of Ardabil City.

## Method

### The study setting and participants

This cross-sectional study was conducted among the market sellers and guilds of Ardabil City in 2023. Due to the high volume of physical contact in the market and the possibility of transmission of infectious diseases, especially COVID-19, marketers and guilds were selected as the target group in this study. According to statistics, there are 14,000 licensed guild units in this city. Considering the heterogeneity of the statistical population, the number of samples was determined using Cochran’s formula. The required sample size was 375, considering d = 0.05 and Z = 1.96.

$$n\, = \,{{{{{Z^2} - pq} \over {{d^2}}}} \over {1 + {1 \over N}\left( {{{{z^2}pq} \over {{d^2}}} - 1} \right)}}$$

Considering a 10% margin of error, the estimated number of participants required for the study was 400. A random cluster sampling method was used, and the city’s main markets were selected as the primary clusters. Sampling was then conducted from each cluster using a random number table. The inclusion criteria were being active as a marketer, having an economic activity license, the shop being active, and the desire to participate in the research. The exclusion criteria included the participants’ unwillingness to participate in the study. Data were collected using a questionnaire that the researcher designed. The data collection process was done after explaining the study’s objectives and obtaining informed consent from the patients. According to paragraphs 7, 25, and 26 of the Helsinki Declaration, to respect the research units, it was not necessary to write the name and surname of the research units when completing the questionnaire. Four hundred people participated in this study. Among the participants, 202 (50.5%) were single, and 198 (49.5%) were married. Also, 316 people (79%) lived in urban areas and 84 (21%) lived in rural areas. In terms of education level, 76 people (19%) had high school, 132 people (33%) had a diploma, 136 people (34%) had an associate’s and bachelor’s degree, and 56 people (14%) had a master’s degree.

### Measurements and scoring

The questionnaire consisted of three parts: 6 questions about demographic variables, five questions on health behaviors during COVID-19, and a TTM researcher-made scale. The TTM scale in the present study had four sub-constructs: (1) stages of change, (2) processes of change, (3) self-efficacy, and (4) decisional balance. Stages of change were assessed using five questions to determine readiness and measure people’s progress toward change regarding adopting preventive health protocols for COVID-19 (Precontemplation, Contemplation, Preparation, Action, and Maintenance). These questions were evaluated using a two-point scale (yes-no). For example, to assess the precontemplation stage, I do not follow the preventive protocols of COVID-19 and do not think about following them. To evaluate the action stage, I have been following the health protocols for preventing COVID-19 for less than six months. The process of change was assessed using 30 questions that were rated on a five-point Likert scale, ranging from “never” (score of 1) to “always” (score of 5). These questions assessed how a person’s cognition and behavior regarding past experiences in the previous month could influence their adherence to health protocols for preventing COVID-19. The process of change includes ten processes, for each of which three questions were designed. We also evaluated self-efficacy using six questions that gauged an individual’s ability to overcome potential obstacles and resist the temptation to neglect health protocols for preventing COVID-19. The assessment was conducted on a five-point Likert scale, ranging from “1” (very low) to “5” (very high). The decisional balance was evaluated through 10 questions that explored an individual’s decision-making process regarding adopting preventive health protocols for COVID-19. These questions illustrated how a person decides to use or abstain from preventive health measures in various situations. The decisional balance includes two sub-structures: benefits (Pros) and disadvantages (Cons); each was evaluated with five questions. Responses were rated on a five-point Likert scale, ranging from “not very important” with a score of 1 to “very important” with a score of 5 [23–25].

Questionnaire validations were conducted using the Content Validation Index (CVI) and Content Validation Ratio (CVR). The questionnaire was administered to a panel of experts comprising ten health education specialists, three infectious disease specialists, and two epidemiologists. The CVI scores for the stages of change, process of change, self-efficacy, and decisional balance were 87%, 96%, 88%, and 90%, respectively. The CVR scores for these dimensions were 88%, 90%, 80%, and 78%, respectively. In addition, using Cronbach’s alpha coefficient, the internal consistency of the self-efficacy subscales, processes of change, and decisional balance was confirmed with values of 0.891, 0.732, and 0.701.

### Data analysis

The data were analyzed using SPSS version 20. Descriptive statistics, including mean and standard deviation tests, were employed to examine the demographic characteristics and scores derived from the model’s constructs. An analysis of variance test was conducted to assess the relationship between the model variables and demographic factors. We used linear regression to determine predictors of the stage of change as a dependent variable, where self-efficacy, processes of change (behavioral Process of change and experiential process of change), and decisional balance (Pros and Cons) were independent variables. The histogram and its results confirmed the normality of the data. The scatter plot also confirmed a correlation and linear relationship between the dependent and independent variables. Durbin Watson’s (DW) statistic showed no autocorrelation between the data (DW = 1.865).

## Result

In this study, 400 marketers from Ardabil city with a mean age of 32.02 ± 6.39 years participated. Half of the people have used a mask for at least a year to prevent COVID-19, and most people (276, 69%) have followed the pattern of hand washing (20–60 s). Table 1 provides more details on preventive behaviors of COVID-19.

**Table 1:** Frequency of preventive behaviors of COVID-19

Variables	Ranges	Frequency	Percentage
History of mask use	Under 3 month	78	19.5
	3–6 month	86	21.5
	6–12 month	36	9.0
	More than 1 year	200	50.0
Hand washing	Less than 20 s	62	15.5
	20–60 s	276	69.0
	More than 60 s	62	15.5
Quality of washing	Only water	22	5.5
	Water soap	188	47.0
	Water and other detergents	190	47.5
Mask location	On the mouth	70	17.5
	Mouth and nose	132	33.0
	Under chin	94	23.5
	In the pocket or bag	104	26.0

The study’s results showed that the majority of the participants did not adhere to the health protocols for the prevention of COVID-19. As shown in Table 2, the majority of the participants (65.5%) were in the passive stages of behavior change (precontemplation, contemplation, and preparation), and only 34.5% were in the active stages, including action and maintenance.

**Table 2:** Stages of change of adherence to health protocols participants

Stages of change	Frequency	Percent
Precontemplation	82	20.5
Contemplation	76	19.0
Preparation	104	26.0
Action	74	18.5
Maintenance	64	16.0
Total	400	100.0

The Pearson correlation coefficient results showed that the transtheoretical model’s sub-constructs are highly correlated. Pros were positively related to the stages of change, while Cons had a negative but non-significant relationship with stages of behavior change. (See Table 3)

**Table 3:** Correlation matrix of structures of transtheoretical model

Variables	SOC	Experiential *P*	Behavioral *P*	Cons	Pros
Experiential P	0.508^**^	-			
Behavioral P	0.550^**^	0.911^**^	-		
Cons	-0.079	-0.463^**^	-0.416^**^	-	
Pros	0.719^**^	0.696^**^	0.621^**^	-0.456^**^	-
Self-efficacy	0.362^**^	0.362^**^	0.352^**^	-0.223^**^	0.337^**^

^**^Correlation is significant at the 0.01 level (2-tailed)

The mean scores of the process of change showed that the behavioral process of change, such as stimulus control, self-liberation, and reinforcement management, played a greater role in keeping samples in the highest level of compliance with health protocols. The post hoc test showed that the mean scores of the process of change in the maintenance and action stages were higher than all the passive stages. (See Table 4)

**Table 4:** Evaluating the progress of stages of change based on the processes of change to follow covid-19 protocols

Process of change	Stages of change*					F	Post hoc
	PC	C	*P*	A	M		
	M ± SD	M ± SD	M ± SD	M ± SD	M ± SD		
Consciousness raising	6.31 ± 2.3	9.7 ± 2.8	10.8 ± 2.3	10.8 ± 2.5	11.6 ± 2.6	55.2	M > A,P, C,PC A, P > C,PC
Dramatic relief	6.02 ± 3.1	10.6 ± 3.09	9.3 ± 2.7	11.29 ± 3	11.34 ± 2.7	44.32	M > A,P, PC, C A > P, C, PC
Environmental Reevaluation	7.2 ± 2.5	10.7 ± 2.7	10.7 ± 2.8	11.2 ± 3	11.1 ± 2.3	29.65	M, A > P,C, PC
Self-reevaluation	6.4 ± 3	10.1 ± 2.3	11.03 ± 2.3	11.6 ± 2.9	12.4 ± 2.6	58.13	M > A,P, C,PC
Social liberation	7.9 ± 3.4	9.7 ± 1.9	10.8 ± 3.3	11.8 ± 2.4	12 ± 2.7	27.32	M > A,P, C,PC
Helping relationships	6.9 ± 2.5	8.4 ± 1.9	10.7 ± 3.2	9 ± 2.6	10.2 ± 3.4	25.16	M > P,C, PC P > A,C, PC
Self-liberation	6.36 ± 3.24	9.6 ± 2.64	11 ± 2.8	11.1 ± 3	12.9 ± 2.3	56.95	M > A,P, C,PC
Counterconditioning	6.9 ± 2.4	10.4 ± 3	10.6 ± 2.4	10.9 ± 2.5	12.1 ± 2.24	45.85	M > A,P, C,PC A > C,PC ; C > PC
Reinforcement management	6.2 ± 2.7	10.8 ± 3	10.5 ± 2.4	12.1 ± 2.9	12.2 ± 2.2	66.43	M > A,P, C,PC A > P,C, PC
Stimulus control	6.5 ± 3	10.3 ± 3.7	10.7 ± 3.2	11.6 ± 3	13 ± 1.9	47.52	M > A,P, C,PC A > P,C, PC

*PC: Precontemplation, C: Contemplation, P: Preparation, A: Action, M: Maintenance

The results of linear regression showed that Pros (β = 0.133, *P* < 0.001), behavioral processes of change (β = 0.058, *P* < 0.001), experimental processes of change (β = 0.053, *P* < 0.001), and self-efficacy (β = 0.007, *P* < 0.001) were the significant predictors of improvement of stage of change to follow health protocols. It should be noted that Cons did not play an important role in the regression model (See Table 5).

**Table 5:** Strongest predictor of compliance with health protocols

Structures of TTM	Unstandardized Coefficients		Standardized Coefficients	T	Sig.
	B	Std. Error	Beta		
Pros	0.133	0.008	0.693	16.240	0.001
Behavioral Process of change	0.058	0.007	0.643	7.847	0.001
Experiential process of change	0.053	0.008	0.585	6.653	0.001
Self-efficacy	0.007	0.002	0.129	3.771	0.001

Dependent Variable: SOC

## Discussion

This study aimed to determine the factors that affect the stages of change or adherence to health protocols among marketers and guilds based on the transtheoretical model (TTM). Findings revealed that most participants were in the inactive stages (pre-contemplation, contemplation, and preparation), while only 34.5% of the samples were in the active stages (action and maintenance) of adhering to the health protocols for preventing COVID-19. We did not find a study that similarly addressed the stages of behavior change in compliance with health protocols during the COVID-19 pandemic. However, some other studies show similar results in the distribution of people in active and passive stages of behavior change. For example, Kamran et al. (2023) reported that the distribution of students in the active phases of physical activity and healthy eating during COVID-19 was 38.3% and 45.4%, respectively [17]. A study in Japan has indicated that more than 60% of people are in the completely inactive stages of pre-contemplation and contemplation about increasing physical activity during the COVID-19 pandemic [18]. In a systematic review based on the results of two studies, Anagaw et al. evaluated TTM as a successful framework for predicting health promotion behaviors and vaccination against COVID-19. Due to the limited evidence, unfortunately, they also did not provide more information about the application and achievements of TTM in the COVID-19 pandemic and preventive behaviors [12].

The basic point is to understand the necessity and importance of determining the stages of behavior change. This study’s results show that most participants did not follow preventive health protocols. Those 65% in the three inactive stages (pre-thinking) need targeted educational intervention to become aware of the subject, receive knowledge, and be stimulated to change behavior. Prochaska believes that change means phenomena that occur over time; therefore, determining the stages of behavior change based on specific times is necessary to develop targeted educational interventions [26].

However, what causes people to move through different stages is a set of factors such as processes of change, decisional balance, and self-efficacy. In the present study, the mean scores of cognitive and behavioral processes of change in the maintenance and action stages were higher, indicating that participants successfully overcame objections to compliance with health protocols (Cons). These findings are consistent with the results reported in a study conducted by Wollesen et al. [27]. Prochaska considers processes of change as the driving force for people to climb the stages of change [26].

In the present study, it is also evident that people in higher stages have obtained higher scores in behavioral and experimental processes. Therefore, to upgrade people from precontemplation and contemplation stages to more active stages, interventions should focus on processes of change. Interventional studies based on TTM have well shown the impact of changes in processes on the promotion of change stages [28–30].

We found that all sub-constructs within the transtheoretical model had a significant relationship with each other. Notably, the Cons sub-construct of decisional balance (representing opinions against the decision to follow health protocols) did not demonstrate a substantial relationship with the stages of change. These findings are consistent with the results obtained in studies conducted by Sacco et al. [31] and Khani Jeihooni et al. [32]. The balance of deciding to perform a healthy behavior is achieved when a person overcomes negative thoughts by overcoming negative feelings and correctly understanding risk.

Self-efficacy was also one of the factors affecting the stages of behavior change, although its effect was weaker than the behavioral processes, Pros, and experimental processes. In a study by Kordi-Kalaki et al. (2022), self-efficacy directly affected performing recommended preventive behaviors for COVID-19 in Iranian adults [33].

Using an extended parallel process model, Yoon et al. (2022) showed that self-efficacy has strong effects on personal hygiene behaviors and social distancing to prevent COVID-19 [34].

Kamran et al. (2023) showed that the self-efficacy score related to physical activity and healthy nutrition in precontemplators was significantly lower than in the active stages (action and maintenance). This study shows that having more self-efficacy is an effective factor in placing people in higher stages of change and adopting health-promoting behaviors [17].

Despite the numerous studies on the effect of self-efficacy on preventive behaviors, Rui et al. (2021) in China concluded that self-efficacy did not have a positive effect on preventive behaviors against COVID-19. They attributed this lack of association to the non-specific measurement scale of their study [35].

A proper preparedness plan is very important for the community’s well-being during crises and disasters. Without such planning, rumors can prevail and overshadow positive thoughts. Ultimately, this can reduce the overall perception of risk in the community and potentially lead to non-compliance with health plans during disasters. [11] This result is based on many studies that have confirmed the effective role of risk perception and risk communication in promoting preventive behaviors against COVID-19 [36–40].

### Limitation

The reliance on a self-report questionnaire imposes constraints on the external validity of this study and may introduce bias in the responses from the research samples. Additionally, the extensive number of questions in Prochaska’s model served as another limitation in this study, as participants during the pandemic showed less interest in responding to lengthy questionnaires.

## Conclusion

The results of this study showed that most marketers in Ardabil City are in the passive stages of observing health protocols. Therefore, due to the many interactions and communications this group has with people, it is necessary to design and implement educational interventions to promote preventive behaviors in them. These interventions can benefit from the effective factors identified as significant in this study, such as behavioral and experimental processes and self-efficacy. Meanwhile, marketers’ risk perception can be considered by improving their understanding of the benefits of following health protocols and overcoming the disadvantages.

## Data Availability

No datasets were generated or analysed during the current study.
